# Biological characterization of compounds from *Rhinella schneideri* poison that act on the complement system

**DOI:** 10.1186/s40409-015-0024-9

**Published:** 2015-08-13

**Authors:** Fernando A. P. Anjolette, Flávia P. Leite, Karla C. F. Bordon, Ana Elisa C. S. Azzolini, Juliana C. Pereira, Luciana S. Pereira-Crott, Eliane C. Arantes

**Affiliations:** Department of Physics and Chemistry, School of Pharmaceutical Sciences of Ribeirão Preto, University of São Paulo (USP), Avenida do Café, s/n, Ribeirão Preto, 14.040-903 SP Brazil; Department of Clinical Analyses, Toxicology and Food Sciences, School of Pharmaceutical Sciences of Ribeirão Preto, University of São Paulo (USP), Avenida do Café, s/n, Ribeirão Preto, 14.040-903 SP Brazil

**Keywords:** Complement system, Hemolytic activity, Neutrophil chemotaxis, *Rhinella schneideri*, Terminal complement complex (SC5b-9), Toad poison

## Abstract

**Background:**

The skin secretions of toads of the family Bufonidae contain biogenic amines, alkaloids, steroids (bufotoxins), bufodienolides (bufogenin), peptides and proteins. The poison of *Rhinella schneideri*, formerly classified as *Bufo paracnemis*, presents components that act on different biological systems, including the complement system. The aim of this study was to isolate and examine the activity of *Rhinella schneideri* poison (*Rs*P) components on the complement system.

**Methods:**

The components active on the complement system were purified in three chromatographic steps, using a combination of cation-exchange, anion-exchange and gel filtration chromatography. The resulting fractions were analyzed by SDS-PAGE and screened for their activity in the hemolytic assay of the classical/lectin complement pathways. Fractions active on the complement system were also assessed for their ability to generate C3 fragments evaluated by two dimensional immunoelectrophoresis assay, C3a and C5a by neutrophil chemotaxis assay and SC5b-9 complex by ELISA assay.

**Results:**

The fractionation protocol was able to isolate the component S5 from the *Rs*P, as demonstrated by SDS-PAGE and the RP-FPLC profile. S5 is a protein of about 6000 Da, while S2 presents components of higher molecular mass (40,000 to 50,000 Da). Fractions S2 and S5 attenuated the hemolytic activity of the classical/lectin pathways after preincubation with normal human serum. Both components stimulated complement-dependent neutrophil chemotaxis and the production of C3 fragments, as shown by two-dimensional immunoelectrophoresis. S2 showed a higher capacity to generate the SC5b***-***9 complex than the other fractions. This action was observed after the exposure of normal human serum to the fractions.

**Conclusions:**

This is the first study to examine the activity of *Rs*P components on the complement system. Fractions S2 and S5 reduced the complement hemolytic activity, stimulated complement-dependent neutrophil chemotaxis and stimulated the production of C3 fragments, indicating that they were able to activate the complement cascade. Furthermore, fraction S2 was also able to generate the SC5b-9 complex. These components may be useful tools for studying dysfunction of the complement cascade.

## Background

The family Bufonidae, with more than 590 species distributed among 50 genera, is one of the largest Anuran families [1]. The genus *Rhinella* is composed of 88 species, of which 36 are found in Brazil [1]. *Rhinella schneideri*, previously known as *Bufo paracnemis*, is the species most commonly encountered in Brazil [2, 3].

Amphibian skin secretions contain a large number of biologically active compounds that are involved in the regulation of physiological functions of the skin, as well as in defense mechanisms against predators and microorganisms [4]. The skin glands produce mucus, peptides, biogenic amines, steroids, and alkaloids. Pharmacologically, these substances may be neurotoxic, cardiotoxic, hemotoxic or myotoxic, and can provoke anesthetic, hypotensive and/or hypertensive effects [5, 6].

Dried poison from the skin glands of the Chinese toad (*Bufo bufo gargarizans cantor*) has been used as a therapeutic agent in traditional Chinese medicine, as well as in other Asian countries [7–9]. Isolated components from toad glands have been used to treat several types of cancer [10–15]. A previous report described the influence of *Rhinella schneideri* poison (*Rs*P) on the lytic activity of the complement system [16].

The complement system (CS) is one of the main defense mechanisms of vertebrates and encompasses over 30 proteins, some of which circulate in the plasma as precursors. Depending on the stimulus, complement activation occurs by classical, alternative or lectin pathways (CP, AP and LP, respectively), leading to a cascade of component interactions and the generation of products that can exert biological activities such as anaphylaxis, chemotaxis, opsonization, immune complex solubilization and participation in the immune response. After recognition, a series of serine proteases is activated, culminating in formation of the “membrane attack complex” (MAC) within the membrane that leads to lysis or cell activation. Two important mediators of the inflammatory reaction, C3a and C5a, are produced as a consequence of CS activation. However, inappropriate activation can result in substantial injury. To prevent undesired complement activation, inhibitors acting at different stages of the activation pathways are used. Despite the large number of inhibitory compounds identified so far, there is still a need for selective complement system modulators [17–19].

Since the poisonous secretion of the parotoid gland of the *R. schneideri* toad presents anticomplement activity, this work aimed to purify the active components and to investigate their effects on the complement system [16].

## Methods

### Poison

The poison was collected by applying pressure to parotoid glands of *Rhinella schneideri* toads, immediately desiccated under vacuum and stored at –20 °C until usage. Prior to the assays, the poison or toxin solutions were filtered through sterilizing membranes (Merck-Millipore, Germany – cellulose ester filters: 0.45 μm and 0.22 μm, respectively).

### Experimental animals

An adult male sheep from the animal facility of the University of São Paulo in Ribeirão Preto was kept in accordance with the ethical guidelines established by the Brazilian College of Animal Experimentation (COBEA). All experiments were approved and conducted in accordance with the ethical principles in animal experimentation adopted by the Ethics Commission for the Use of Animals (CEUA), Campus of Ribeirão Preto, USP (protocol n^o^ 05.1.637.53.6).

### Fractionation of *R. schneideri* poison

The soluble material from the desiccated poison (500 mg) was clarified by filtration through membranes (0.45 μm and then 0.22 μm, Merck-Millipore, Germany). The material was chromatographed at 4 °C on a 2.5 × 63.0 cm column of CM-cellulose-52 (Whatman, USA), which was equilibrated and initially eluted with 300 mL of 0.05 M NH_4_HCO_3_ buffer, pH 7.8, when a convex concentration gradient was started from 0.05 to 1.00 M NH_4_HCO_3_ buffer. Fractions of 3.0 mL were collected. Absorbance at 280 nm and buffer concentration profiles were then traced as previously described [20].

The resulting pools, designated C1 to C7, were then lyophilized until salt-free. The fraction C1 showed the lowest percentage of hemolysis. Therefore, C1 was submitted to the next fractionation step. The soluble material from fraction C1 (56.6 mg in 5 mL of 0.05 M Tris–HCl, pH 7.8, centrifuged at 15,700 *× g*, at 4 °C, for 10 min) was applied on a 1.0 × 10.0 cm DEAE-Sepharose column at room temperature, previously equilibrated with 0.05 M Tris–HCl, pH 7.8 (buffer A). Elution was performed with a linear gradient of buffer B (0.05 M Tris–HCl supplemented with 1.0 M NaCl, pH 7.8), at a flow rate of 0.5 mL/min. Absorbance was monitored at 280 nm. The chromatography was performed in an Äkta™ Prime system (GE Healthcare, Sweden) and the resulting pools, denominated D1 to D4, were lyophilized. Fraction D3 showed the highest activity on the complement system and was submitted to a molecular filtration on a Sephacryl S-200 column (1.6 cm × 60 cm) at room temperature, previously equilibrated with PBS (phosphate buffered saline), pH 7.4, at a flow rate of 0.4 mL/min. The absorbance was monitored at 254 nm. The resulting pools, designated S1 to S5 were grouped according to their respective absorbance peaks and stored at –20 °C.

Fractions S2 and S5, which showed activity on the complement system, were submitted to a reversed phase FPLC using a C2C18 column (0.46 × 10 cm, Amersham Biosciences, Sweden). The column was equilibrated with 0.1 % (V/V) trifluoroacetic acid (TFA, solution A); and the components were eluted by a step concentration gradient from 0 to 100 % of solution B (80 % acetonitrile, 0.1 % trifluoroacetic acid, V/V), at a flow rate of 0.5 mL/min, at room temperature. The absorbance (λ = 214 nm) was registered by the Äkta™ Prime system (GE Healthcare, Sweden).

### Polyacrylamide gel electrophoresis

Sodium dodecyl sulfate polyacrylamide gel electrophoresis (SDS-PAGE) was run as described by Laemmli [21]. The gel was stained with Silver Staining Kit Protein (Pharmacia Biotech, Sweden) or Coomassie Blue R-350. Conditions of voltage and amperage (maximum values: 90 V, 40 mA and 15 W) were controlled by an EPS 3500 XL Electrophoresis Power Supply (Pharmacia Biotech, Sweden).

### Solutions

Cells were washed in PBS, pH 7.4, and complement fixation diluent (CFD) containing 0.1 % gelatin (gel) was used for hemolytic assays of CP/LP activity as described by Harrison and Lachmann [22]. Modified Alsever’s solution [23] was used as an anticoagulant for sheep blood storage.

### Normal human serum (NHS) and erythrocytes

Human blood was obtained from healthy donors (approval certificate by the Research Ethics Committee – CAAE, protocol n° 0022.0.212.000–08). Blood samples were collected from healthy volunteers of both sexes (aged 20 to 30 years) without anticoagulant and allowed to clot for one hour at room temperature, after which they were centrifuged at 556 × *g,* for ten minutes at 4 °C, and the NHS obtained was stored at –70 °C.

Healthy adult male sheep were bled by jugular vein puncture; the blood was collected in two volumes of Alsever’s modified solution, stored at 4 °C and utilized for 15 days as a source of erythrocytes for CP/LP hemolytic assays. Sheep blood was centrifuged (556 × *g*, 15 min, 4 °C), after which the plasma and buffy coat were discarded. Red cells were washed twice in PBS, suspended in CFD/Gel and mixed with an appropriate volume of anti-sheep erythrocyte antibody. This erythrocyte-antibody suspension was maintained at 4 °C for 15 min and its absorbance at 700 nm was adjusted to 0.70–0.80.

### Hemolytic complement assay

NHS was diluted in CFD/Gel at a ratio of 1:20, V/V. Fractions (100 μL in PBS, S1 – A_280_ ~ 0.35; S2 – A_280_ ~ 0.20; S3 – A_280_ ~ 0.17; S4 – A_280_ ~ 0.10 and S5 – A_280_ ~ 0.16) obtained from D3 molecular filtration (Sephacryl S-200) were incubated with CFD/Gel solution (12.5 μL) and diluted serum (1:20; 37.5 μL) for one hour at 37 °C. After the incubation period, erythrocyte-antibody suspension (100 μL) was added to the samples and a new incubation was performed for 30 min at 37 °C. At the end of the incubation, cold PBS (250 μL) was added to the samples, which were then centrifuged at 556 × *g* for ten minutes. The percentage of lysis was determined by the absorbance at 412 nm, using as 100 % lysis control the suspension of lysed erythrocytes in water, and as 0 % lysis control the cells incubated in CFD/Gel. The positive control was prepared under the same reaction conditions except that the fraction volume was replaced by PBS (100 μL). This assay was employed to monitor the activity of the fractions on the complement system during the purification process.

### Human neutrophils suspension

Human blood from healthy donors was mixed with modified Alsever’s solution (V/V) and centrifuged at 978 × *g* for ten minutes. Neutrophils were isolated by the gelatin method, as described by Paula *et al.* [24] with modifications. Briefly, after blood centrifugation, the plasma and buffy coat were discarded, and the cell pellet was suspended in two volumes of 2.5 % gelatin solution prepared in 0.15 M NaCl. This suspension was incubated for 15 min at 37 °C. After incubation, the upper neutrophil-rich layer was collected, diluted in 30 mL of 0.15 M NaCl solution and centrifuged at 757 × *g* for ten minutes at room temperature. The cell pellet was suspended in 20 mL of 0.83 % NH_4_Cl solution, pH 7.8, and incubated for five minutes at 37 °C, in order to lyse remaining erythrocytes. After incubation, the supernatant was discarded and the suspension was centrifuged at 757 × *g* for ten minutes at room temperature. The cell pellet was washed in 30 mL of 0.15 NaCl solution and centrifuged at 757 × *g* for ten minutes. The supernatant was discarded and 1 mL of the neutrophil suspension was suspended in 1 mL of Hank’s solution containing 0.1 % gelatin. Cells were diluted (1:10) in Turk solution and counted in a Neubauer Chamber. The neutrophil purity of 80–90 % with viability higher than 95 % was accomplished by trypan blue exclusion test. One neutrophil suspension was standardized to contain 1.2 x 10^6^ cells mL^−1^ and used in the neutrophil chemotaxis assay.

### Neutrophil chemotaxis assay

The chemotaxis assay was performed using a modified version of Boyden’s technique [25], in which 120 μL of NHS with 50 μL of CFD/Gel and 50 μL of each fraction, S1 (A_280_ ~ 0.35), S2 (A_280_ ~ 0.20), S3 (A_280_ ~ 0.17), S4 (A_280_ ~ 0.10) and S5 (A_280_ ~ 0.16), obtained from molecular filtration of D3, were placed in the lower migration chamber and covered with a filter of 13 mm diameter and 3 μm pore (SSWP 01300, Merck-Millipore, Germany). The upper compartment of the chamber was filled with 300 μL of a suspension of human neutrophils (1.2 × 10^6^ cells mL^−1^). Next, all chambers were closed and incubated at 37 °C for 60 min in a humid atmosphere. After incubation, the filters were removed from the chambers, fixed in propanol, stained with Harris hematoxylin, dehydrated in isopropanol and cleared with xylene. Each filter was placed between a slide and a coverslip with Entellan (Merck KGaA, Germany). A mixture of NHS (120 μL) with CFD/Gel (100 μL) and zymosan (75 μL, 1 mg/mL) was used as positive control, and NHS (120 μL) with CFD/Gel (100 μL) as negative control.

Neutrophil migration within the filter was determined under light microscopy by the leading-front method, measuring in micrometers the greatest distance crossed by three cells per field [26]. At least ten fields were examined at 100× magnification for each filter.

### Two-dimensional immunoelectrophoresis (2D-IEP)

For this analysis, 50 μL of fractions S2 (A_280_ ~ 0.2) and S5 (A_280_ ~ 0.16) were preincubated in a water bath with 100 μL of NHS 1:2 by 60 min at 37 °C. Immunoelectrophoresis was performed according to the method of Clark and Freeman [27], using glass plates (5.5 × 7.5 × 0.2 cm) and 1.3 % agarose in buffer (0.025 M Tris–HCl, 0.027 M glycine, 0.02 M sodium barbital, 0.01 M EDTA, pH 8.8). In the first dimension, the positive control (31.25 μL of zymosan plus 100 μL of 1:2 NHS), the negative control (100 μL of 1:2 NHS plus 50 μL of PBS) and fraction S2 and S5 (50 μL of each fraction plus 100 μL 1:2 NHS) were electrophoresed for four hours, at 140 V and 5 mA/plate. For the second dimension, the plates were completed with 1.3 % agarose (5 mL) containing 1 % anti-human C3 antibody (Calbiochem/Merck, Germany) and electrophoresed for 14 h, at 10 W and 5 mA/plate. The plates were dried at room temperature, stained with Ponceau 0.5 % and destained with 10 % acetic acid.

### Evaluation of the capacity to generate the SC5b-9 complex

The capacity of fractions (S1 to S5) to generate the SC5b-9 complex was evaluated by enzyme-linked immunosorbent assay (ELISA, Quidel SC5b-9 Complement® kit, USA) after exposure of the NHS to 50 μL of each fraction [28].

### Statistical analysis

The results were expressed as the mean ± SEM. The groups were compared statistically by ANOVA followed by the Tukey post-hoc test. All data were analyzed via Prism™ v.5 (GraphPad Inc., USA).

## Results

### Fractionation of *R. schneideri* poison

**Fig. 1:**
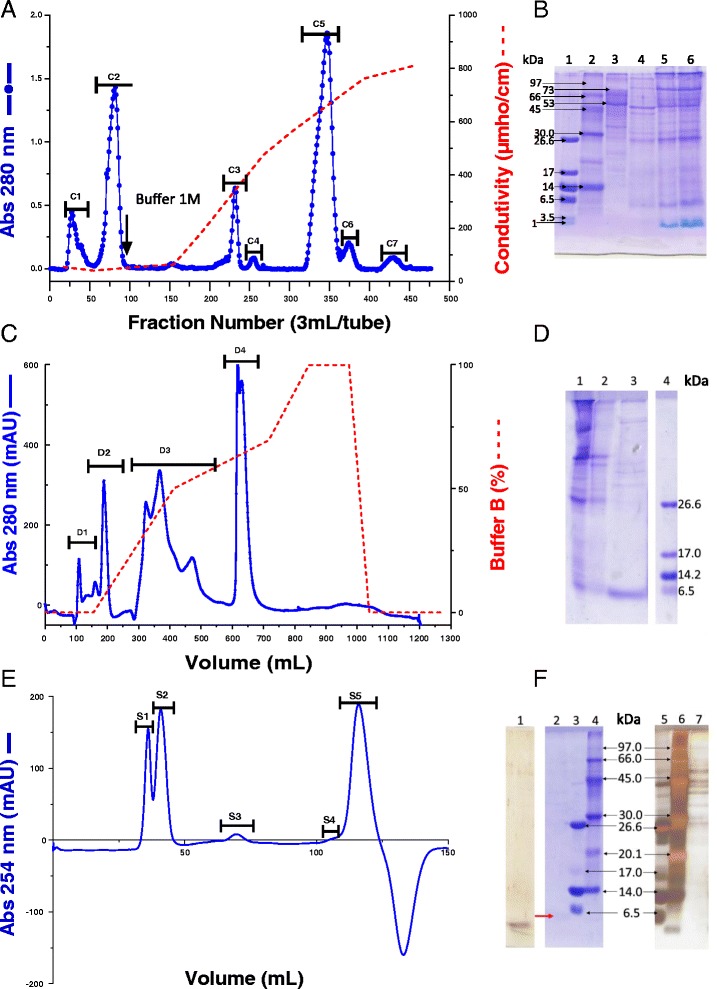
Fractionation of *Rhinella schneideri* poison (*Rs*P). **a** Chromatographic profile of *Rs*P on CM-cellulose-52. The column was equilibrated with 0.05 M ammonium bicarbonate, pH 7.8. The sample (extract from 500 mg) was applied at a flow rate of 20 drops/min; and adsorbed /components were eluted using a convex concentration gradient of NH_4_HCO_3_ (0.05 to 1.0 M, pH 7.8). Fractions (3.0 mL/tube) were collected at 4 °C. **b** SDS-PAGE using 13.5 % separation gel. Lanes 1, 2 and 3 – ultra- mass markers, respectively. Lane 4 – fraction C1; lanes 5 and 6 – *Rs*P. **c** Chromatographic low (Sigma-Aldrich, USA), low-high (GE Healthcare, Sweden) and high (GE Healthcare, Sweden)molecular profile of fraction C1 on DEAE-Sepharose. The column was equilibrated with 0.05 M Tris–HCl, pH 7.8 (buffer A). The sample (56.6 mg of C1) was applied at a flow rate of 0.5 mL/min; and adsorbed components were eluted using a linear gradient from 0–1 M NaCl in equilibrating buffer (buffer B). Elution with 100 % buffer B was achieved after 150 mL. **d** SDS-PAGE using 13.5 % separation gel. Lane 1 – *Rs*P; lane 2 – fraction C1; lane 3 – fraction D3; lane 4 – ultra-low molecular mass markers (Sigma-Aldrich, USA). **e** Chromatographic profile of fraction D3 on Sephacryl S-200. The column, equilibrated with PBS, pH 7.4, was eluted with this same buffer (flow rate: 0.4 mL/min) and fractions of 1 mL were collected. In (**a**) and (**c**), the elution profiles were monitored at 280 nm, while in (**e**) the profile was monitored at 254 nm. **f** SDS-PAGE using 13.5 % separation gel. Lanes 1 and 2 – fraction S5; lanes 3 and 5 – ultra-low-molecular-mass markers (Sigma-Aldrich, USA); lanes 4 and 6 – low-molecular-mass markers (GE Healthcare, Sweden); lane 7 – fraction S2

The components from *Rhinella schneideri* poison with activity on the CS were obtained by three chromatographic steps: cation-exchange, anion-exchange and molecular exclusion. The chromatographic profile of soluble poison on CM-cellulose-52 (cation-exchange) showed seven different fractions, denominated C1 to C7 (Fig. 1a). Fraction C1 presents the highest inhibition of hemolytic complement activity, as previously demonstrated by our group [29]. *Rs*P and fraction C1 were assayed by SDS-PAGE (Fig. 1b), where C1 appeared as a complex mixture of proteins. Therefore, it was submitted to the next fractionation step on a DEAE-Sepharose column (Fig. 1c).

Among the four fractions (D1, D2, D3 and D4) obtained from the rechromatography of fraction C1, the fraction D3 presented the highest activity on CS [29]. Unfortunately, it was composed of low- and high-molecular-mass components, highlighting a protein with an approximate molecular mass of 6 kDa, observed in the SDS-PAGE (Fig. 1d). In order to isolate some component presenting action on complement system, fraction D3 was submitted to a gel filtration on a Sephacryl-S200 column (Fig. 1e). Five fractions, designated S1 to S5, were obtained; and the active fractions S2 and S5 were analyzed by SDS-PAGE (Fig. 1f). Fraction S2 was composed of high-molecular-mass proteins (40,000 to 50,000 Da), while S5 was a protein of about 6 kDa. The recoveries of chromatographic fractions with activity on CS are shown in Table 1.

**Table 1:** Recovery of the chromatographic components obtained during the fractionation procedure

Fraction	Fractionation step	Fraction recovery^a^ (%)
*Rs*P	–––	100.0
C1	CM-cellulose-52	7.6
D3	DEAE-Sepharose	4.1
S2 and S5	Sephacryl-S100	1.2 (S2) and 2.1(S5)

^a^Total recovery was calculated based on the area under the peak by UNICORN software (GE Healthcare, Sweden)

**Fig. 2:**
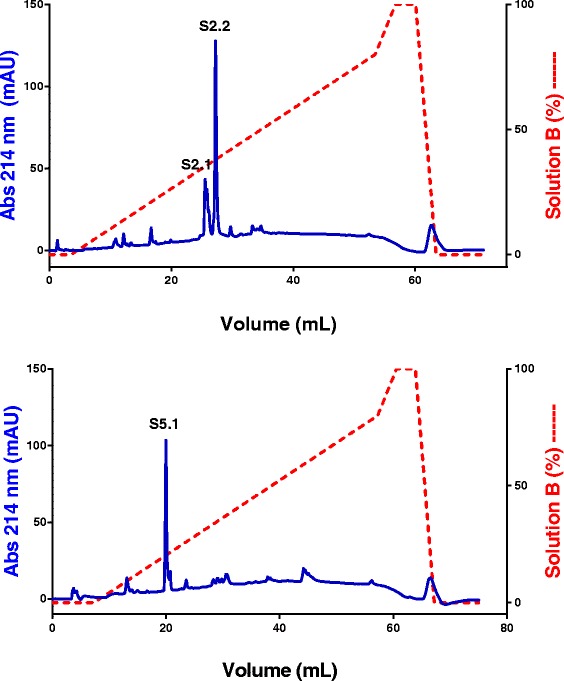
Reversed-phase FPLC of fractions S2 and S5. The C2C18 column was equilibrated with 0.1 % (V/V) trifluoroacetic acid (TFA, solution A). Adsorbed proteins were eluted using a concentration gradient from 0 to 100 % of solution B (80 % acetonitrile in 0.1 % TFA, V/V). Fractions of 0.5 mL/tube were collected at a flow rate of 0.5 mL/min

The active fractions S2 and S5 were submitted to a reversed phase FPLC using a C2C18 column (Fig. 2). S5 showed higher purity than S2, which presented a chromatographic profile with two major peaks, S2.1 and S2.2.

### Hemolytic complement assay

**Fig. 3:**
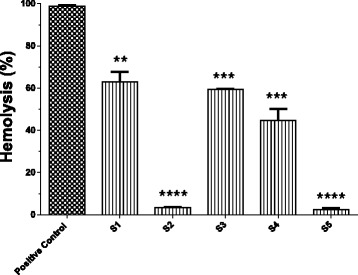
Effect of fractions S1 - S5 on the classical/lectin hemolytic pathways of complement activation. The fractions (100 μL in PBS, S1 – A_280_ ~ 0.35; S2 – A_280_ ~ 0.20; S3 – A_280_ ~ 0.17; S4 – A_280_ ~ 0.10 and S5 –A_280_ ~ 0.16)) were incubated for one hour at 37 °C with normal human serum diluted 1:20 (37.5 μL) and CFD/Gel solution (12.5 μL). The positive control was run under the same conditions but in the absence of fractions. The absorbances of supernatants from cells incubated in CFD/Gel buffer (0 % lysis) and lysed in water (100 % lysis) were employed to calculate the percentage of lysis induced by NHS in the absence (positive control) or presence of the fractions (tests). The columns represent means ± SEM of an experiment performed in duplicate. ** *p* < 0.01, *** *p* < 0.001 and **** *p* < 0.0001 compared to the positive control

All fractions obtained from the last chromatographic procedure were subjected to the hemolytic assay of the classical/lectin pathway, in which 100 μL volumes of each fraction – S1 (A_280_ ~ 0.35), S2 (A_280_ ~ 0.2), S3 (A_280_ ~ 0.17), S4 (Ab_280_ ~ 0.1) and S5 (A_280_ ~ 0.16) – were used. The hemolytic activities of the classical/lectin pathways observed in the presence of all fractions were significantly lower than the positive control, particularly in the presence of fractions S2 and S5 (Fig. 3).

### Two-dimensional immunoelectrophoresis

**Fig. 4:**
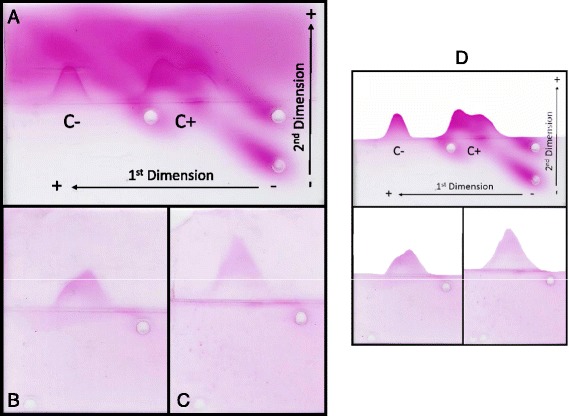
Immunoelectrophoretic analysis of C3 in NHS incubated with fractions S2 and S5. **a** Positive control (C+) with zymosan (31.25 μL, 1 mg/mL) and NHS (100 μL, 1:2), and negative control (C-) with PBS (50 μL) and NHS (100 μL, 1:2). **b** Fraction S2 (50 μL, A_280_ ~ 0.2) with SHN (100 μL, 1:2). **c** Fraction S5 (50 μL, A_280_ ~ 0.16) with NHS (100 μL, 1:2). All mixtures were incubated for 60 min in a water bath at 37 °C. The plates were dried at room temperature, stained with 0.5 % Ponceau and bleached with 10 % acetic acid. Electrophoretic conditions: first dimension – four hours, 140 V at 15 mA and 10 W; second dimension – 14 h at 15 mA and 10 W. **d** Figures were manipulated to remove the background highlighting the presence of one peak for C- and two peaks for C+, S2 and S5 assays

The 2D-IEP profile of the positive control shows two protein peaks, corresponding to C3 and C3b, indicating the ability of zymosan to activate the complement system, leading to partial cleavage of C3 (Fig. 4a). A symmetrical peak was observed in the negative control, corresponding to intact C3. The 2D-IEP profiles obtained in the presence of S2 and S5 (Fig. 4b and c, respectively) also showed two peaks, similar to the positive control, corresponding to C3 and C3b, indicating that S2 and S5 were able to activate the complement system. The background of these 2D-IEP profiles (Fig. 4a, b and c) were removed in order to highlight the presence of one peak for negative control and two peaks for positive control, S2 and S5 assays (Fig. 4d).

### Neutrophil chemotaxis assay

**Fig. 5:**
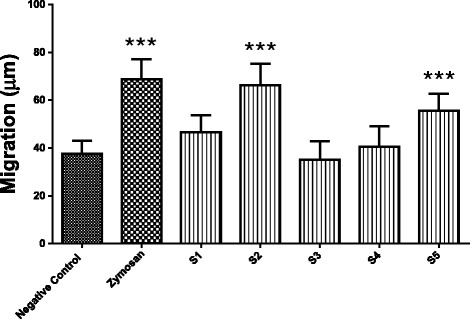
Neutrophil chemotaxis induced by normal human serum (NHS) preincubated with fractions S1 - S5. The fractions were preincubated with NHS for 60 min at 37 °C. The positive control consisted of 120 μL of NHS with 100 μL of CFD/Gel buffer and 75 μL of zymosan (1 mg/mL), while the negative control was 120 μL of NHS with 100 μL of CFD/Gel buffer. Neutrophil migration was assessed by the leading-front technique, in which at least ten microscopic fields were analyzed per filter at 100× magnification. The columns represent the means ± SEM of one experiment done in triplicate. *** *p* < 0.001 compared to the negative control

A significant (*p* < 0.001) increase in neutrophil migration was observed by preincubation of poison components S2 and S5 with NHS (Fig. 5). These results indicate that S2 and S5 were able to induce activation of the complement system, leading to the formation of chemotactic factors.

### Evaluation of the capacity to generate the SC5b-9 complex

**Fig. 6:**
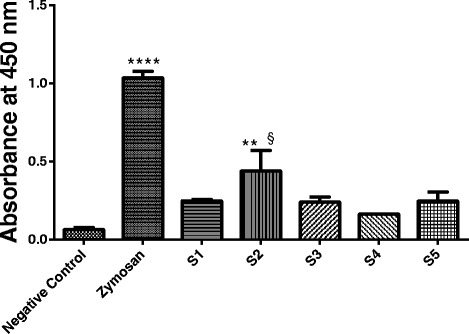
Formation of the SC5b-9 complex. NHS was incubated for 60 min with PBS (negative control), zymosan (positive control; 1 mg/mL) and fractions S1 to S5. The assay was done using a commercial kit (Quidel SC5b-9 Complement® kit, USA). The columns represent the means ± SEM of one experiment done in duplicate. ** *p* < 0.01 and **** *p* < 0.0001 compared to the negative control, and § *p* < 0.05 compared to S4

The concentrations of SC5b-9 complex produced after exposure to NHS with fractions S1, S2, S3, S4 and S5, as well as zymosan (positive control) were determined by enzyme-linked immunosorbent assay (Fig. 6). S2 showed significant capacity to generate the SC5b-9 complex compared to the negative control (*p* < 0.01) and § compared to S4 (*p* < 0.05).

## Discussion

Research involving animal-derived substances that act on the complement system has been well documented in the literature. Spider (*Loxosceles*), snake (Elapidae, Crotalidae and Viperidae), honeybee, wasp and scorpion venoms have demonstrated the ability to activate the CS [30–35]. This activation can be initiated by cleavage of a specific component or by interaction with other CS components resulting in the formation of the “membrane attack complex” [32]. *Tityus serrulatus* venom activates the CS, leading to factor B and C3 cleavage, reduction of serum lytic activity and generation of complement chemotactic factors [30]. Assis *et al*. [16] showed that fractionation of the poisonous secretion of *B. marinus paracnemis* Lutz (currently named *Rhinella schneideri*), by dialysis and chromatography on QAE-Sephadex, yielded a fraction with anticomplementary activity when incubated with human serum. This effect was evaluated by measuring the kinetics of lytic activity on sensitized sheep red blood cells (classical pathway) and unsensitized rabbit cells (alternative pathway). A study of the skin secretion of six species of common toads in China revealed that only *Bombina maximum* poison showed direct hemolytic activity at a dose of 20 μg/mL [36].

This study describes the effects of two components from *Rs*P that interfere with CP/LP of the complement system. To the best of our knowledge, only one study reported the interaction of *Rs*P with the CS until now [16]. The ability of this poison to induce serum-related leukocyte recruitment was evaluated as an indicator of complement activation and consequent generation of complement chemotactic factors.

The procedure used in this study to fractionate the active compound from *Rs*P was relatively simple, involving only three chromatographic steps, a cationic and an anionic chromatography followed by a gel filtration. The SDS-PAGE results show that fraction C1, active on CS, is composed of high- and low-molecular-mass proteins. The major protein of fraction D3 has a molecular mass of about 60,000 Da and corresponds to the isolated S5 active component. On the other hand, the fraction S2 is composed mainly of high-molecular-weight proteins (40,000–50,000 Da). The chromatographic profile of the RP-FPLC S5 confirms the high purity of this component.

The complement activation occurs along classical, alternative or lectin pathways leading to a cascade of component interactions and generation of products that present such biological activities as anaphylaxis, chemotaxis, opsonization, immune complex solubilization, participation in the immune response and other activities [17–19]. Two important mediators of the inflammatory reaction, C3a and C5a, are produced as a consequence of CS activation [17–19].

The hemolytic complement assay was used to ensure the functional integrity of the whole pathways (classical or alternative) with the terminal pathway. The results obtained showed that all fractions (S1 – A_280_ ~ 0.35, S2 – A_280_ ~ 0.2, S3 – A_280_ ~ 0.17, S4 – Ab_280_ ~ 0.1 and S5 – A_280_ ~ 0.16) induced significant reductions in hemolytic activity of classical/lectin pathways, but smaller hemolysis values were obtained in the presence of S2 and S5 (*p* < 0.0001). The solutions of the fractions (S1-S5) used in the hemolytic assay showed different absorptions at 280 nm. Contrary to what might be expected in this context, our objective was to perform only a qualitative analysis of the effect of the fractions on the CS. The fraction solutions with the highest possible concentration were used, considering their solubility as well as the amount obtained from each fraction (S3 and S4 are present in low proportion in the fraction D3 – Fig. 1e).

We have chosen to use the absorbance at 280 nm and the volume as a means to quantify the samples, because toad poison is composed of proteic (protein and peptides) and non-proteic (mucus, biogenic amines, steroids and alkaloids) compounds. The non-proteic compounds interfere with many protein quantification assays, invalidating the measurement of the sample. According to Marongio [29], the protein concentration, determined by the biuret method, of a dispersion of 5 mg/mL of the *R. schneideri* poison was only 1.32 mg/mL, corresponding to 26 % of the total weight of the poison.

The reduction in CP/LP lytic activity induced by S2 and S5 suggests an activation of the complement cascade during the preincubation phase (NHS + fractions) and subsequent inactivation (unstable components). The CS activation preceding the addition of red blood cells would reduce serum concentrations of complement components, thereby leading to decreased NHS lytic activity during the hemolytic assay. Similar results were observed in studies of snake venoms from the genera *Bothrops* (*B. jararaca, B. moojeni* and *B. cotiara*), *Micrurus* (*M. ibiboboca* and *M. spixii*) and *Naja* (*N. naja, N. melanoleuca* and *N. nigricollis*) [32, 37, 38].

The presence of an inhibitor of CS in *Rs*P is also possible, since protease inhibitors have been identified on the skin of *Anura* species [39–41]. Several compounds can modify or interact with CS by activating or inhibiting it [16, 30–38, 42]. Peptides synthesized from phage-displayed peptide libraries based on C1q binding are able to inhibit the hemolytic activity of the classical complement pathway [43]. Another peptide from phage-displayed libraries, the compstatin peptide, a 13-amino-acid cyclic peptide, binds to a β-chain of C3 and inhibits activation of both the classical and alternative pathways [44, 45].

The immunoelectrophoresis assay showed that cleavage of C3 in serum incubated with S2 and S5 (Fig. 4b and c, respectively), was similar to that induced by incubation of NHS with zymosan (positive control, Fig. 4a), corroborating the hypothesis that poison components induce activation of the CS. Bertazzi *et al*. [30] showed that *Tityus serrulatus* venom was also able to alter C3 immunoelectrophoresis migration after incubation with NHS.

The chemotaxis assay served as an indicator of the activation of CS and consequent generation of neutrophil chemoattractant factors. S2 and S5 increased neutrophil migration by interacting with CS components, leading to subsequent cleavage of C3 and C5, which produced the active fragments C3a and C5a (anaphylatoxins) during the preincubation phase (60 min at 37 °C) of NHS with fractions. These results were similar to that presented by zymosan (positive control) and confirm that S2 and S5 are able to activate the complement system. A similar effect was observed in a prior study on *Tityus serrulatus* venom [30]. BaP1, a 24 kDa metalloprotease isolated from *Bothrops asper* venom, induced neutrophil chemotaxis that was mediated by agents derived from activation of the complement system [37, 46].

The assay performed to evaluate the capacity of the *Rs*P components to induce SC5b-9 complex formation showed that only S2 was able to induce a significant increase (*p* <0.01) in the SC5b-9 concentration, compared to negative control (Fig. 6). This assay was conducted to better clarify the action of fractions on the complement system and provided an additional indication of the terminal complement system activation induced by S2.

Primary among the effects of SC5b-9 complex is tissue injury through cell lysis or stimulation of pro-inflammatory mediators [47]. It is known that more than 80 % of C5a and SC5b-9 is generated by activation of the mannose-binding lectin or classical pathway [48, 49]. High levels of activation and generation of SC5b-9 complex are related to several pathological states, including lupus erythematosus and rheumatoid arthritis [47].

Proteolytic activity evaluation of the fractions S2 and S5 was performed using chromogenic substrate for alpha-chymotrypsin (Sigma-S7388, N-Succinyl-Ala-Ala-Pro-Phe p-nitroanilide, Sigma-Aldrich, USA) and for coagulation proteases [Sigma-T6140, N-(p-Tosyl)-Gly-Pro-Lys 4-nitroanilide acetate salt, Sigma-Aldrich, USA]. Additionally, these fractions were subjected to assays to evaluate inhibitory activity against trypsin and chymotrypsin proteases. Neither fraction showed proteolytic or inhibitory activity (data not shown), indicating that their actions on the CS are not by proteolysis or inhibition of the complement cascade proteases.

Several approaches are being proposed for the development of new pharmacological agents directed at diseases in which the CS is active [47, 50–53]. Cobra venom factor (CVF) is a non-toxic venom compound with functional and structural characteristics very similar to the complement component C3 [53, 54]. The development of the humanized version of CVF is a promising therapeutic agent for many pathologies [50, 52, 53].

## Conclusion

In summary, our results indicate that *Rs*P presents components, especially S2 and S5, that are able to activate the complement cascade, as evidenced by the decreased serum lytic activity, production of C3 fragments, increased leukocyte migration and SC5b-9 generation. Based on these findings, the *Rs*P may be considered a rich source of substances that can be used as molecular tools to study dysfunction of the CS, since they are able to modulate the activity of this system.

### Ethics committee approval

All experiments were approved and conducted in accordance with the ethical principles in animal experimentation adopted by the Ethics Commission for the Use of Animals (CEUA), Campus of Ribeirão Preto, USP (protocol n^o^ 05.1.637.53.6). The use of human blood was approved by the Research Ethics Committee of the School of Pharmaceutical Sciences of Ribeirão Preto, University of São Paulo (USP) under protocol n° 0022.0.212.000–08.

## Abbreviations

AP: Alternative pathway
CFD: Complement fixation diluents
CP/LP: Classical/lectin pathway
CVF: Cobra venom factor
FPLC: Fast protein liquid chromatography
Gel: Gelatin
CS: Complement system
2D-IEP: Two-dimensional immunoelectrophoresis
MAC or SC5b-9: Membrane attack complex
NHS: Normal human serum
PBS: Phosphate-buffered saline
*Rs*P: *Rhinella schneideri* poison
SDS-PAGE: Sodium dodecyl sulfate polyacrylamide gel electrophoresis
TFA: Trifluoroacetic acid
